# Structural and functional roles of ether lipids

**DOI:** 10.1007/s13238-017-0423-5

**Published:** 2017-05-18

**Authors:** John M. Dean, Irfan J. Lodhi

**Affiliations:** Division of Endocrinology, Metabolism and Lipid Research, Department of Medicine, Washington University School of Medicine, Saint Louis, MO 63110 USA

**Keywords:** ether lipids, plasmalogen, phospholipid, peroxisomes, cancer, metabolic disorders

## Abstract

Ether lipids, such as plasmalogens, are peroxisome-derived glycerophospholipids in which the hydrocarbon chain at the *sn-1* position of the glycerol backbone is attached by an ether bond, as opposed to an ester bond in the more common diacyl phospholipids. This seemingly simple biochemical change has profound structural and functional implications. Notably, the tendency of ether lipids to form non-lamellar inverted hexagonal structures in model membranes suggests that they have a role in facilitating membrane fusion processes. Ether lipids are also important for the organization and stability of lipid raft microdomains, cholesterol-rich membrane regions involved in cellular signaling. In addition to their structural roles, a subset of ether lipids are thought to function as endogenous antioxidants, and emerging studies suggest that they are involved in cell differentiation and signaling pathways. Here, we review the biology of ether lipids and their potential significance in human disorders, including neurological diseases, cancer, and metabolic disorders.

## INTRODUCTION

**Figure 1:**
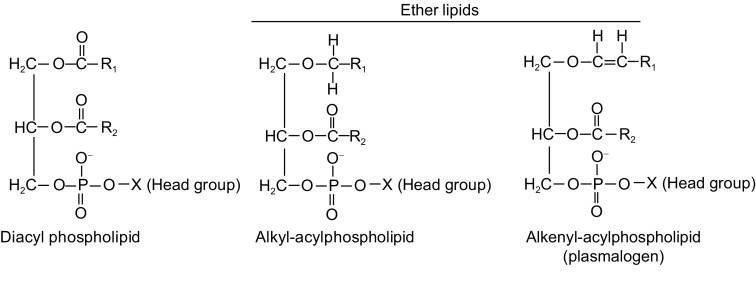
Chemical structures of diacyl and ether-linked phospholipids. Diacyl phospholipids have fatty acyl side chains linked to the *sn-1* and *sn-2* position of the glycerol backbone by ester bonds. Ether-linked phospholipids are a subclass of glycerophospholipids that have an alkyl chain attached by an ether bond at the *sn-1* position. The *sn-2* position of ether lipids generally has an ester-linked acyl chain, as in diacyl phospholipids. Some ether-linked phospholipids, called alkenyl-acylphospholipids, contain a *cis* double bond adjacent to the ether linkage and are commonly referred to as plasmalogens. The polar head group of ether-linked phospholipids is most commonly choline or ethanolamine

Conventional glycerophospholipids have acyl chains attached to the *sn-1* and *sn-2* positions of the glycerol backbone by ester bonds. Ether lipids are a unique class of glycerophospholipids that have an alkyl chain attached to the *sn-1* position by an ether bond (Fig. 1). The alcohol moiety attached to the phosphate group in ether lipids is generally choline or ethanolamine, but occasionally inositol or serine have also been observed. Ether lipids constitute approximately 20% of the total phospholipid pool in mammals, but tissue distribution varies. The highest levels are found in the brain, heart, spleen, and white blood cells, while liver has scant amount of intracellular ether lipids (Braverman and Moser, 2012).

Plasmalogens are the most common form of ether lipids and are characterized by a *cis* double bond adjacent to the ether linkage. Plasmalogens were serendipitously discovered in 1924 by Feulgen and Voit while staining tissue sections with a nuclear stain that reacts with aldehydes released by acid hydrolysis of DNA (Snyder, 1999). Because the acid treatment also resulted in breakdown of the vinyl ether bond of plasmalogens to generate aldehydes, the researchers unexpectedly also observed cytoplasmic staining and named the unknown source of aldehydes “plasmalogens”. The study of plasmalogens and ether lipids in general has been long and arduous and in the beginning the progress was slow and scattered. Recently, much advancement has been made in our understanding of the roles of ether lipids in health and disease.

In this review, we outline the pathway of ether lipid biosynthesis and then describe the unique structural roles played by ether lipids in mammalian cell membranes, followed by a discussion of emerging cellular functions of these lipids. Lastly, we review the current knowledge of the role of ether lipid metabolism in disease.

## BIOSYNTHESIS OF ETHER LIPIDS

**Figure 2:**
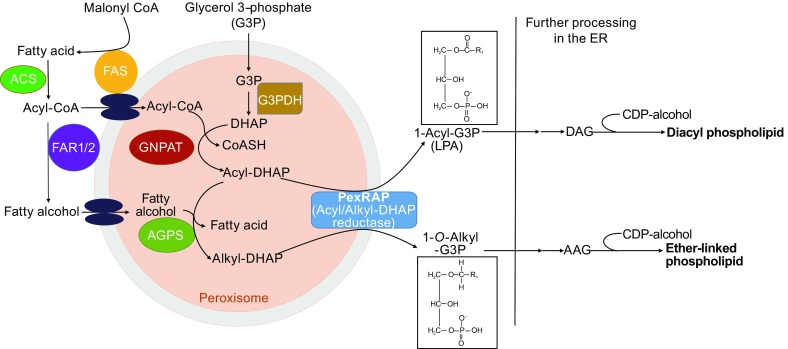
Acyl-DHAP pathway of ether lipid synthesis. This process begins in peroxisomes and is subsequently completed in the ER. The pathway utilizes dihydroxyacetone phosphate (DHAP) generated by glycerol 3-phosphate dehydrogenase (G3PDH)-mediated dehydrogenation of G3P as the substrate for ether lipid synthesis. Fatty acid synthase (FAS)-mediated *de novo* lipogenesis generates fatty acyl-CoA that is utilized by glyceronephosphate O-acyltransferase (GNPAT) or reduced to a fatty alcohol by a fatty acyl-CoA reductase (FAR1 or FAR2) to later be catalyzed by alkylglycerone phosphate synthase (AGPS), forming the ether bond and exchanging the acyl chain for an alkyl group. PexRAP (an acyl/alkyl DHAP reductase) then catalyzes the final peroxisomal step, generating the ether lipid precursors, 1-*O*-alkyl-G3P (AGP) or the diacyl phospholipid precursor lysophosphatidic acid (LPA). The completion of phospholipid synthesis occurs in the ER. This includes acylation of the glycerol backbone at the *sn-2* position, converting LPA to diacylglycerol (DAG) and AGP to alkyl-acylglycerol (AAG), as well as addition of a cytidine diphosphate-alcohol head group (such as CDP-choline or CDP-ethanolamine) at the *sn-3* position to form the mature phospholipid

Ether lipids are synthesized through a well-characterized process that begins in the peroxisome and is completed in the ER (Fig. 2). The peroxisomal component is referred to as the acyl-dihydroxyacetone (DHAP) pathway since DHAP, a glycolysis intermediate, is used as a precursor for ether lipid synthesis (Hajra and Das, 1996). Fatty acids for ether lipid synthesis are thought to be derived from fatty acid synthase (FAS)-mediated *de novo* lipogenesis or dietary sources (Lodhi et al., 2012, 2015). FAS, a large multifunctional enzyme that primarily synthesizes palmitate (C16:0), is partially localized to the peroxisomes, where it interacts with various peroxisomal proteins (Hillebrand et al., 2012; Lodhi et al., 2012; Cader et al., 2016). In order to be used for ether lipid synthesis, fatty acids are first activated to fatty acyl-CoAs by an acyl-CoA synthetase (ACS) associated with the peroxisomal membrane. Glyceronephosphate O-acyltransferase (GNPAT), a peroxisomal matrix protein, uses a long chain acyl-CoA to acylate DHAP at the *sn-1* position. The next peroxisomal step is catalyzed by alkylglycerone phosphate synthase (AGPS) and results in the formation of the ether bond at the *sn-1* position by exchanging an acyl chain for an alkyl group. The alkyl moiety used in the AGPS-catalyzed step is generated by a peroxisomal membrane-associated fatty acyl-CoA reductase (FAR1 or FAR2), which reduces an acyl-CoA to a fatty alcohol. The final peroxisomal step in ether lipid synthesis is carried out by acyl/alkyl-DHAP reductase, which reduces alkyl-DHAP into the ether lipid precursor 1-*O*-alkyl glycerol-3-phosphate (AGP). The DHAP reductase can also reduce acyl-DHAP to generate the diacyl phospholipid precursor lysophosphatidic acid (LPA) as an alternative to direct acylation of glycerol-3-phosphate (G3P) by a G3P acyltransferase (GPAT) (Hajra and Das, 1996). Further steps to convert AGP or LPA to corresponding ether-linked or diacyl glycerolipids, such as phosphatidylcholine or phosphatidylethanolamine, take place in the ER (Hajra and Das, 1996; Gibellini and Smith, 2010). Tethering of peroxisomes to the ER for completion of lipid synthesis was recently shown to involve interaction of the peroxisomal protein acyl-CoA-binding domain 5 (ACBD5) with VAMP-associated proteins A and B (VAPA and VAPB), ER-resident proteins that recruit organelles to the ER (Hua et al., 2017). The precise mechanism through which the ACBD5-VAP complex permits exchange of lipids between the organelles is unclear.

Although the acyl/alkyl-DHAP reductase protein was purified from guinea pig liver and biochemically characterized over 40 years ago (LaBelle and Hajra, 1974), the gene encoding this enzyme in mammals remained unknown. We recently identified this gene as *dhrs7b* and renamed the protein PexRAP (for peroxisomal reductase activating PPARγ) based on its proposed function in adipose tissue (Lodhi et al., 2012). Unlike inactivation of GNPAT (also called DHAP acyltransferase), which results in almost complete loss of ether lipids (Rodemer et al., 2003), knockout of PexRAP in mice results in 50–80% decrease in the ether lipid levels (Lodhi et al., 2015). Similarly, approximately 50% decrease in ether lipids was observed in a mutant of CHO-K1 cells with almost complete absence of acyl/alkyl DHAP reductase activity isolated in an ethyl methanesulfonate (EMS) mutagenesis screen (James et al., 1997), suggesting that there is a salvage pathway of ether lipid synthesis. The precise nature of this salvage pathway is poorly understood, but is thought to involve dephosphorylation of alkyl-DHAP to alkyl-dihydroxyacetone, followed by its reduction to alkyl-glycerol, which upon phosphorylation can re-enter the biosynthetic pathway downstream of acyl/alkyl-DHAP reductase (James et al., 1997).

## BIOLOGICAL FUNCTIONS OF ETHER LIPIDS

Ether lipids are chemically distinct from their diacyl counterparts, allowing them to contribute unique structural characteristics to biological membranes, which affect such factors as membrane fluidity and membrane fusion. In addition to these effects on membrane dynamics, studies with *in vitro* and *in vivo* models of ether lipid deficiency suggest that ether lipids are involved in a variety of biological functions, including regulating cell differentiation, impacting cellular signaling, and reducing oxidative stress through their ability to function as potential endogenous antioxidants.

### Structural roles

Ether lipids are a major structural component of cell membranes. The incorporation of ether-linked alkyl chains in phospholipids alters their physical properties and affects membrane dynamics. This is largely attributed to the lack of a carbonyl oxygen at the *sn-1* position, which facilitates stronger intermolecular hydrogen bonding between the headgroups (Lohner, 1996). Moreover, the vinyl-ether linkage of plasmalogens at the *sn-1* position allows the proximal regions of the *sn-1* and *sn-2* chains to become parallel, favoring close alignment (Han and Gross, 1990; Paltauf, 1994), therefore permitting tighter packing of phospholipids in the membrane resulting in decreased membrane fluidity and increased rigidity. This property is particularly important in higher order membrane structures such as those found in myelin, evidenced by its enrichment in plasmalogens (Farooqui and Horrocks, 2001). Both ether lipid-deficient mouse models and human patients with ether lipid deficiency often display defects in myelination in both the central and peripheral nervous systems (da Silva et al., 2012).

Plasmalogens are enriched in lipid raft microdomains (LRM), cholesterol-rich regions of membranes where many cellular signaling proteins are concentrated (Pike et al., 2002; Rodemer et al., 2003). Recently, lipidomic analysis of rat synaptic membranes during the first 60 days of postnatal development showed dynamic changes in membrane lipids, including the progressive accumulation of plasmalogens along with cholesterol and sphingolipids. Reconstitution of this membrane remodeling in a model membrane system was associated with enhancement of raft domain stability (Tulodziecka et al., 2016). Consistent with the requirement of plasmalogens in the organization of LRM, plasmalogen-deficient GNPAT knockout mice display disrupted lipid raft formation and mislocalization of cholesterol to a perinuclear compartment (Rodemer et al., 2003).

**Figure 3:**
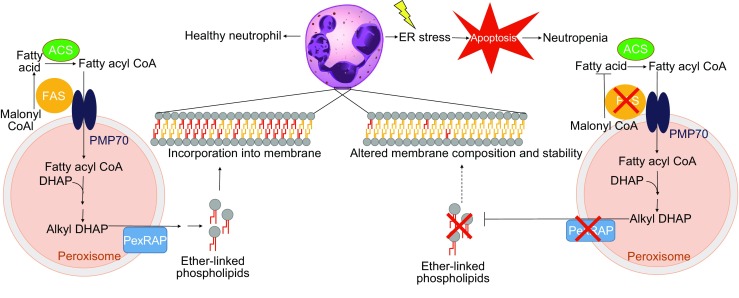
Peroxisomal lipid synthesis is required for maintaining neutrophil viability and membrane integrity. In healthy neutrophils, fatty acid synthase (FAS) and PexRAP participate in peroxisomal lipid synthesis, including the generation of ether-linked phospholipids that are incorporated into the plasma membrane, contributing to membrane integrity. Genetic ablation of FAS or PexRAP disrupts the lipid synthetic pathway, leading to a preferential loss of ether-linked phospholipids in neutrophils. This alters membrane stability, leading to the activation of ER stress, increased apoptosis, and ultimately neutropenia

Disruption of cellular phospholipid composition affects membrane integrity (Li et al., 2006), activates ER stress (Fu et al., 2011; Volmer et al., 2013), and promotes apoptosis (Yen et al., 1999). It has long been known that ether-linked phospholipids are abundantly present in neutrophils, constituting nearly half of the phosphatidylcholine pool (Mueller et al., 1982, 1984; Bräutigam et al., 1996; Nagan and Zoeller, 2001). However, the physiological significance of these lipids in neutrophils has remained unclear. Our recent studies with genetic inactivation of lipogenesis in mice suggest that ether lipids are an important membrane component required for survival of mature neutrophils (Fig. 3). Tamoxifen-inducible global knockout of FAS results in a preferential decrease in ether-linked glycerophosphocholines (ether-lipid equivalent of phosphatidylcholine; GPC) in neutrophils (Lodhi et al., 2015), increased ER stress and apoptosis, resulting in nearly complete neutropenia, suggesting that FAS channels fatty acids to peroxisomes for lipid synthesis. Indeed, FAS has been shown to interact with PMP70, an ATP binding cassette subfamily D transporter that transports fatty acyl CoA into peroxisomes (Hillebrand et al., 2012), directly linking FAS to peroxisomal lipid metabolism. Consistent with the importance of peroxisome-derived lipids in neutrophil survival, tamoxifen-inducible knockout of PexRAP also results in neutropenia due to decreased ether lipid levels, recapitulating the results obtained with FAS knockout (Lodhi et al., 2015). These results suggest that peroxisome-derived lipids, including ether lipids, are an important structural component of neutrophil membranes and reveal a novel lipogenic pathway that selectively regulates neutrophil development.

### Membrane trafficking

Model membranes enriched in plasmalogens have a greater tendency to form non-lamellar inverse hexagonal structures at lower temperatures as compared to such membranes composed of diacyl phospholipids (Glaser and Gross, 1994). Since a transition from a lamellar (no curvature) to an inverted hexagonal (negative curvature) phase is required for membrane fusion (Marrink and Mark, 2004), this property of plasmalogens has been linked to their role in facilitating processes of membrane trafficking. Notably, synaptic vesicles are enriched in ether lipids, and neurotransmitter release from these vesicles into the presynaptic cleft is impaired in the synaptosomes (nerve terminals) isolated from the brains of ether lipid deficient mice (Brodde et al., 2012). In further support of the notion that ether lipids facilitate membrane fusion processes, ether lipid deficiency disrupts the integrity of the blood-testis barrier by affecting the sorting, endocytosis, and recycling of tight junction proteins (Komljenovic et al., 2009). Moreover, plasmalogens have been implicated in cholesterol transport. CHO cells with a defect in plasmalogen synthesis have impaired plasma membrane to ER transport and esterification of cholesterol (Munn, 2003). It is unclear whether plasmalogens directly regulate cholesterol transport through effects on membrane dynamics or if a signaling molecule released from hydrolysis of plasmalogens is involved in the process.

### Cell signaling and differentiation

In addition to their structural roles, ether lipids can also act as biological signaling molecules. Oxidation of the vinyl-ether bond of plasmalogens by brominating species produced by an eosinophil peroxidase in activated eosinophils results in a α-bromo fatty aldehyde that acts as a phagocyte chemoattractant for the recruitment of other leukocytes to sites of inflammation (Thukkani et al., 2002; Albert et al., 2003). Moreover, ether-linked mono-alkyl glycerophosphates serve as endogenous antigens to activate invariant natural killer T (iNKT) cells. GNPAT-deficient mice show impaired thymic maturation of iNKT cells and have a reduced overall number of iNKT cells in the thymus and periphery, suggesting that ether lipids are important for the development of these cells (Facciotti et al., 2012).

Ether lipids have also been identified as potential endogenous ligands of peroxisome proliferator-activated receptor gamma (PPARγ) (Davies et al., 2001; McIntyre et al., 2003; Zhang et al., 2004; Tsukahara et al., 2006), a nuclear receptor critical for adipose tissue development and lipid metabolism, which is the target of the glitazone family of diabetes drugs. Using an unbiased mass spectrometry-based approach, we identified several ether-linked GPC species that were bound to PPARγ in a rosiglitazone-displaceable manner. Treatment of cells with one of these lipid species promoted PPARγ-dependent luciferase reporter activity (Lodhi et al., 2012). Furthermore, inhibition of ether lipid biosynthesis by knockdown of PexRAP blocks PPARγ dependent gene expression and adipocyte differentiation. This effect was rescued by rosiglitazone treatment, suggesting that peroxisomal lipid synthesis is involved in endogenous ligand generation (Lodhi et al., 2012). Recently, disruption of peroxisomal biogenesis by Pex16 knockdown was shown to impair PPARγ activation and adipocyte differentiation. The defect in adipogenesis was rescued with the treatment of alkyl glycerol (Hofer et al., 2017), an ether lipid precursor that enters the synthetic pathway downstream of the peroxisomal steps.

**Figure 4:**
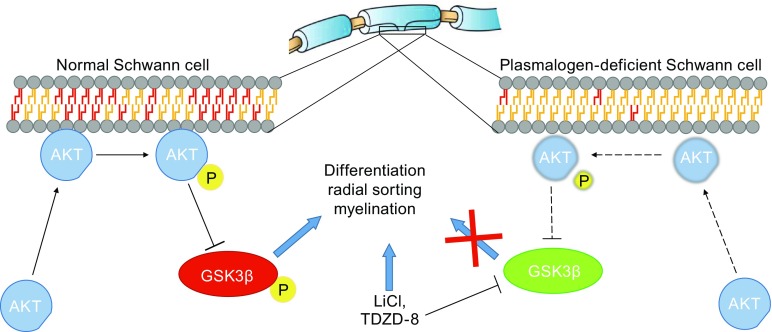
Role of ether lipids in Schwann cell differentiation and myelination. In a healthy Schwann cell, the cell membrane is enriched in plasmalogens. This is necessary for proper recruitment of Akt to the plasma membrane, resulting in its phosphorylation and activation. Activated Akt phosphorylates GSK3β at Ser9, inhibiting its activity. Plasmalogen deficiency impairs recruitment and activation of Akt, preventing it ability to inhibit GSK3β. Active GSK3β impairs Schwann cell differentiation, resulting in disrupted radial sorting and myelination. Supplementation of lithium or TDZD-8 to inhibit GSK3β rescues plasmalogen-deficient phenotype, restoring differentiation and myelination

Ether lipids have also been implicated in the differentiation of other cell types. In the peripheral nervous system, Schwann cells are required for maintaining axonal health and myelination. Impaired ether lipid synthesis is associated with defects in myelination and myelin structure (Brites, 2003; Rodemer et al., 2003; Teigler et al., 2009). Recent studies suggest that this may be related to the requirement of plasmalogens in Schwann cell differentiation and function. Plasmalogen deficiency mediated by GNPAT or Pex7 knockout in mice impairs membrane recruitment and activation of Akt, resulting in hyperactivation of glycogen synthase kinase 3β (GSK3β), which inhibits Schwann cell differentiation (Fig. 4). This in turn impairs axonal sorting and myelination. Inhibition of GSK3β using lithium rescues Schwann cell differentiation, bypassing the effect of plasmalogen deficiency (da Silva et al., 2014). Plasmalogens have also been implicated in Akt signaling in other cell types. Plasmalogens protect against apoptosis induced through serum starvation by modulating Akt signaling, a pathway possibly mediated through plasmalogens’ ability to activate G-protein coupled receptor signaling (Hossain et al., 2013, 2016). Supplementation of Neuro-2 cells with plasmalogens suppress neuronal apoptosis signaling induced via serum starvation (Yamashita et al., 2015a).

### Plasmalogens as cellular antioxidants

Plasmalogen-deficient cultured cells and animals have long been known to be more sensitive to oxidative damage as compared to their wild-type counterparts, setting the basis for the notion that plasmalogens may function as cellular antioxidants (Morand et al., 1988; Zoeller et al., 1988; Reiss et al., 1997). This is thought to be due to the presence of the vinyl ether bond, which makes plasmalogens susceptible to oxidative attacks. In particular, there is evidence to suggest that plasmalogens could protect unsaturated membrane lipids from oxidation by singlet oxygen (Broniec et al., 2011). Plasmalogens may also be involved in scavenging a variety of reactive oxygen species (ROS) (Morand et al., 1988; Khaselev and Murphy, 1999; Maeba et al., 2002; Skaff et al., 2008). Recent studies suggest that myelin in sciatic and optic nerves from plasmalogen-deficient mice lacking the peroxisomal biogenesis factor Pex7 is significantly more susceptible to oxidative damage than the myelin from wild-type mice (Luoma et al., 2015).

The idea that plasmalogens have a protective role against oxidative damage is difficult to validate directly in complex biological systems. Paradoxically, pro-oxidant effects of these lipids have also been reported in a bulk lipid system (Wang and Wang, 2010). A recent study using synthetic choline plasmalogens in model membrane systems suggests that the antioxidant effect of plasmalogens occurs intramolecularly rather than intermolecularly and depends on the degree of unsaturation of the *sn-1* and *sn-2* chains, the length of these chains, and their conformation in membranes (Broniec et al., 2017). This suggests that the antioxidant capacity of plasmalogens may be contextually dependent.

## ETHER LIPIDS IN DISEASE

The critical importance of ether lipids for health in humans is highlighted by the severity of inherited peroxisomal disorders caused by ether lipid deficiency. Emerging studies suggest that altered ether lipid production is also associated with several other disorders including neurodegenerative diseases, cancer, and metabolic disorders. However, the molecular mechanisms underlying the role of ether lipids in these diseases remain unclear for the most part, suggesting a need for future research.

### Genetic peroxisomal disorders resulting in ether lipid deficiency

#### Rhizomelic chondrodysplasia punctata

Rhizomelic chondrodysplasia punctata (RCDP) is a peroxisomal disorder caused by ether lipid deficiency. RCDP is a multisystem, developmental disorder and has a rare occurrence affecting approximately 1/100,000 individuals. Symptoms include not only those that give the disease its namesake, shortening of limbs (rhizomelia) and multiple punctate epiphyseal calcification (chondrodysplasia punctata), but also microcephaly, distinct facial features, cataracts, and psychomotor retardation. The disease is fatal with patients rarely surviving past the tenth year of life (Braverman and Moser, 2012; White et al., 2003). The most common form is RCDP type 1, which is caused by mutations in Pex7, a peroxisomal import receptor for proteins, including AGPS, that contain a type 2 peroxisomal targeting sequence (PTS), resulting in defects in peroxisomal biogenesis (Braverman et al., 1997; Motley et al., 1997; Purdue, 1997). The less common RCDP types 2 and 3 are caused by single enzyme mutations in either the GNPAT or AGPS genes, respectively, and result in deficient ether lipid biosynthesis. Recently, two new types of RCDP have been reported. RCDP type 4 results from a mutation in FAR1, which is required for the conversion of fatty acyl-CoAs to fatty alcohols (Honsho et al., 2013; Buchert et al., 2014). RCDP type 5 results from a genetic mutation leading to specific loss of only the Pex5 long isoform (Pex5L). While both the long and short Pex5 isoforms recognize PTS1-containing proteins, Pex5L is a co-receptor with Pex7 for the import of PTS2-containing proteins, and its loss results only in impaired PTS2-tagged protein import and RCDP phenotype (Barøy et al., 2015).

#### Zellweger spectrum disorders

Peroxisomal biogenesis disorders in the Zellweger spectrum are a heterogeneous group of genetic disorders encompassing Zellweger syndrome, neonatal adrenoleukodystrophy, and infantile Refsum disease. They are caused by a mutation in one of the dozen Pex genes required for normal assembly and functions of peroxisomes and affect approximately 1 in 50,000 individuals. Mutations in Pex1 are the most common, accounting for nearly 70% of cases. However, mutations in Pex3, Pex16, and Pex19 result in the complete absence of peroxisomes and are coupled to the most severe phenotypes (Steinberg et al., 2012). Like RCDP, Zellweger spectrum disorders (ZSD) present with a broad range of clinical characteristics, which include craniofacial abnormalities, chondrodysplasia punctata, hepatomegaly, kidney abnormalities, and vision problems. Infants with a severe form of ZSD (Zellweger syndrome) have multiple developmental abnormalities and generally die within the first year of life. Along with blood accumulation of very long chain and branched chain fatty acids, which require peroxisomes for their oxidation, decreased ether lipid levels in red blood cell membranes are one of the criteria for diagnosis of ZSD (Braverman et al., 2016).

Generation of mouse models of ether lipid deficiency has shed some light on the pathophysiology of peroxisomal biogenesis disorders. These mice show defects in neuronal development and myelination (Brites, 2003; Rodemer et al., 2003; Teigler et al., 2009). As discussed above, Schwann cells of ether-lipid deficient mice show impaired differentiation and myelination resulting from decreased Akt signaling, presumably due to the necessity of plasmalogens for correct recruitment of Akt to the membrane (da Silva et al., 2014).

### Alterations in ether lipid levels in common disease states

#### Neurological disorders

Alzheimer’s disease (AD) pathology is associated with decreased ether lipids, particularly plasmalogens, in the brain (Ginsberg et al., 1995, 1998; Han et al., 2001; Han, 2005). Additionally, serum levels of ethanolamine plasmalogens in AD patients are decreased and correlate with the severity of disease progression (Goodenowe et al., 2007; Wood et al., 2010). Given this relationship, serum plasmalogen levels have even been used to successfully stratify diseased patients (Wood et al., 2016). Increased β-amyloid (Aβ) levels associated with AD have been shown to promote oxidative stress in the brain, leading to loss of peroxisomal function. This in turn decreases AGPS (the rate-limiting enzyme of ether lipid synthesis) activity, ultimately lowering plasmalogen levels (Grimm et al., 2011). In addition, certain ethanolamine plasmalogen species are thought to have the ability to destabilize existing Aβ fibrils *in vitro* and prevent the formation of new fibrils in a dose dependent manner, providing another mechanism by which plasmalogen loss facilitates AD pathology (Yamashita et al., 2015b).

Abnormal ether lipid levels have also been implicated in other neurological disorders. Red blood cells and fibroblasts collected from schizophrenia patients were recently shown to be significantly decreased in plasmalogen levels (Tessier et al., 2016; Huang et al., 2016). In addition, patients with Parkinson’s disease show severe alterations in neuronal membrane composition, including a significant decrease in postmortem frontal cortex lipid raft plasmalogens levels (Fabelo et al., 2011). Additional work is needed to understand the molecular mechanism through which reduced ether lipids contribute to these pathologies.

#### Cancer

Decades ago it was observed that cancer cells have remarkably higher levels of ether lipids compared to normal cells, suggesting that ether lipids may be involved in cancer pathogenesis (Snyder et al., 1966, 1969; Snyder and Wood, 1969; Albert and Anderson, 1977; Roos and Choppin, 1984). Indeed, increased ether lipids are correlated with more aggressive cancers, and knockdown of AGPS in a variety of cancer cell lines results in impaired pathogenicity (Benjamin et al., 2013). A small molecule screen identified an inhibitor of AGPS that decreases ether lipid levels and appears to reduce pathogenicity of human cancer cells *in vitro* (Piano et al., 2015). Recently, a lipidomics study on breast cancer patients identified increased plasma ether-linked phosphatidylcholine species as a diagnostic marker for breast cancer (Chen et al., 2016). It is unclear whether ether lipids play a structural or signaling role in promoting cancer progression.

#### Metabolic disorders

Levels of plasmalogen and other ether-linked phosphatidylcholine species are increased in steatotic livers of high fat diet-fed mice, although it is unclear whether this is due to increased synthesis, reduced degradation, or impaired hepatic release (Eisinger et al., 2014). One possibility is that increased plasmalogens may be an indication of increased activity of protective mechanisms against oxidative stress (Barr et al., 2012). However, serum plasmalogen levels are decreased in patients with nonalcoholic steatohepatitis (NASH) and nonalcoholic fatty liver disease (NAFLD) as compared to healthy controls (Puri et al., 2009). This drop in plasmalogen levels is more severe in NASH patients as opposed to simple steatosis patients suggesting the possibility to use serum lipidomics as a biomarker to diagnose and stratify the disease. Recent studies suggest a protective role of plasmalogens against NASH. Free cholesterol-induced NASH in mice was associated with decreased levels of docosahexaenoic acid-containing plasmalogens and impaired expression of PPARα, a critical transcriptional regulator of fatty acid oxidation. Rescue of plasmalogen levels by alkyl glycerol treatment prevented hepatic steatosis and NASH through PPARα-dependent increase in fatty acid oxidation, suggesting a role for endogenous plasmalogen production in PPARα signaling (Jang et al., 2017).

Decreased serum ether lipid levels have been implicated in hypertension and obesity (Pietiläinen et al., 2007; Graessler et al., 2009). In a weight-discordant twin study, obese patients showed a decrease in serum plasmalogens compared to healthy twin controls, indicating that the effect is independent of genetic makeup (Pietiläinen et al., 2007). Similarly, a follow up study by the same group showed that plasmalogens containing arachidonic acid at the *sn-2* position are reduced in the adipocyte membranes of morbidly obese twins compared to metabolically healthy twins (Pietiläinen et al., 2011). Conversely, serum plasmalogen levels increase in men with aerobic training and in patients with a healthy dietary intervention (Lankinen et al., 2016; Felder et al., 2017).

Altered ether lipid levels are also associated with type 1 diabetes (T1D). Interestingly, decreased ether lipids were detected in the serum of children that were later diagnosed with T1D. The presence of these metabolic changes prior to the detection of autoantibodies suggests that ether lipid metabolism is dysregulated before the disease emerges (Orešič et al., 2008). It is unresolved whether the decreased ether lipid levels contribute to accelerating pathology of diabetes or are related to secondary metabolic changes. However, β cells are known to possess inherently poor antioxidant capacity (Lenzen et al., 1996), and this coupled with the lower levels of plasmalogens may result in enhanced susceptibility of β cells to metabolic stress and ROS contributing to the autoimmune response. Regardless of the mechanism, changes in lipid metabolism may serve as useful markers for early diagnosis.

## CONCLUDING REMARKS AND FUTURE DIRECTIONS

With the rising interest in lipidomics, ether lipids have emerged as potential biomarkers of several diseases, including neurodegenerative diseases, cancer, and metabolic disorders (Eisinger et al., 2014; Chen et al., 2016; Tessier et al., 2016; Alshehry et al., 2016). However, with this observation comes the principal question: Are the changes in ether lipid levels merely a byproduct of the disease process or a contributor to disease pathogenesis? Severe phenotypes resulting from genetic loss of ether lipid production suggest that these peroxisome-derived phospholipids are responsible for fundamental cellular functions that cannot be carried out by conventional phospholipids. Decreased ether lipid synthesis is linked to multiple neurological and metabolic abnormalities, while increased levels are associated with cancer, suggesting that proper regulation of ether lipid production is required for normal physiology. Future research will be necessary to determine if targeted inhibition of the ether lipid synthetic pathway could treat malignancies. Conversely, there is a need to develop novel dietary interventions to correct pathologies associated with ether lipid deficiency. Several groups have attempted to deliver alkylglycerols in diet to boost ether lipid levels in deficient animals with variable results. While the effects of ether lipid replacement appear to be promising in peripheral tissues, the results have been less successful in correcting neurological deficits (Brites et al., 2011; Rasmiena et al., 2015; Jang et al., 2017). It will be of great interest to develop more effective synthetic ether lipid precursors that could cross the blood-brain barrier and alter the clinical course of neurological diseases.

Currently there is a wide knowledge gap in our understanding of the molecular mechanisms underlying the pathologies associated with altered production of these lipids. Much work has been done using mouse models of global ether lipid deficiency, revealing gross abnormalities associated with such manipulations (da Silva et al., 2012). Going forward it will be important to take a more fine-tuned approach through targeted inactivation of ether lipid production to better define the biological roles of these unique phospholipids, perhaps uncovering novel strategies for disease intervention.
